# Extensive plastome reduction and loss of photosynthesis genes in *Diphelypaea coccinea*, a holoparasitic plant of the family Orobanchaceae

**DOI:** 10.7717/peerj.7830

**Published:** 2019-10-02

**Authors:** Eugeny V. Gruzdev, Vitaly V. Kadnikov, Alexey V. Beletsky, Andrey V. Mardanov, Nikolai V. Ravin

**Affiliations:** 1 Institute of Bioengineering, Research Center of Biotechnology of the Russian Academy of Sciences, Moscow, Russia; 2 Moscow State University, Moscow, Russia

**Keywords:** Parasitic plant, Plastid genome, Orobanchaceae, *Diphelypaea*

## Abstract

**Background:**

Parasitic plants have the ability to obtain nutrients from their hosts and are less dependent on their own photosynthesis or completely lose this capacity. The reduction in plastid genome size and gene content in parasitic plants predominantly results from loss of photosynthetic genes. Plants from the family Orobanchaceae are used as models for studying plastid genome evolution in the transition from an autotrophic to parasitic lifestyle. *Diphelypaea* is a poorly studied genus of the Orobanchaceae, comprising two species of non-photosynthetic root holoparasites. In this study, we sequenced the plastid genome of *Diphelypaea coccinea* and compared it with other Orobanchaceae, to elucidate patterns of plastid genome evolution. In addition, we used plastid genome data to define the phylogenetic position of *Diphelypaea* spp.

**Methods:**

The complete nucleotide sequence of the plastid genome of *D. coccinea* was obtained from total plant DNA, using pyrosequencing technology.

**Results:**

The *D. coccinea* plastome is only 66,616 bp in length, and is highly rearranged; however, it retains a quadripartite structure. It contains only four rRNA genes, 25 tRNA genes and 25 protein-coding genes, being one of the most highly reduced plastomes among the parasitic Orobanchaceae. All genes related to photosynthesis, including the ATP synthase genes, had been lost, whereas most housekeeping genes remain intact. The plastome contains two divergent, but probably intact *clpP* genes. Intron loss had occurred in some protein-coding and tRNA genes. Phylogenetic analysis yielded a fully resolved tree for the Orobanchaceae, with *Diphelypaea* being a sister group to *Orobanche* sect. *Orobanche*.

## Introduction

About 1% of all angiosperm species can parasitize other flowering plants or mycorrhizal fungi (Nickrent et al., 1997). Facultative or obligatory hemiparasites still carry out photosynthesis to some extent, while holoparasites have completely lost the ability to perform photosynthesis, and obtain nutrients from their host. Transition from an autotrophic to heterotrophic lifestyle is associated with a relaxation of selection pressure on photosynthesis-related genes, both in the nuclear and the plastid genomes. Most evident is a functional and physical reduction of the plastid genome (plastome), which correlates with a loss of genes encoding the photosynthetic machinery and related functions, increased substitution rates, and structural rearrangements (DePamphilis & Palmer, 1990; Wolfe, Morden & Palmer, 1992; Barrett et al., 2014). Since the plastome contains both photosynthesis-related and housekeeping genes, gene loss is not random and follows a particular pattern (Barrett & Davis, 2012; Barrett et al., 2014; Graham, Lam & Merckx, 2017). The NAD(P)H dehydrogenase (*ndh*) genes are usually lost first, followed by photosynthesis-related genes (*psa*, *psb*, *pet*, *rbcL* and *atp*) and plastid-encoded RNA polymerase. Housekeeping genes encoding rRNAs, ribosomal proteins and tRNAs are the last to be lost. Extensive studies of parasitic plants have revealed different levels of plastome degradation and their correlation with the types of parasitism, ranging from minimal in hemiparasitic members of Orobanchaceae (Wicke et al., 2013) and Viscaceae (Petersen, Cuenca & Seberg, 2015) to extreme in some holoparasitic species, such as *Pilostyles aethiopica* (Bellot & Renner, 2015), and even possibly complete loss of the plastome, as in *Rafflesia lagascae* (Molina et al., 2014).

The broomrape family, Orobanchaceae, is an ideal lineage to study plastid genome evolution in the course of transition to heterotrophy, since it comprises about 2000 hemiparasitic and holoparasitic species (Bennett & Mathews, 2006; McNeal et al., 2013), along with three autotrophic genera, *Lindenbergia*, *Rehmannia* and *Triaenophora*. Within Orobanchaceae, holoparasitism has evolved independently at least three times (Young, Steiner & dePamphilis, 1999; McNeal et al., 2013). Complete sequences of plastid genomes have been reported for *Lindenbergia philippensis* (Wicke et al., 2013), *Triaenophora shennongjiaensis* (Xia & Wen, 2018), six *Rehmannia* species (Zeng et al., 2017), and photosynthetic hemiparasitic species of the genera *Schwalbea* (Wicke et al., 2013), *Pedicularis* (Cho et al., 2018), *Castilleja* (Fan et al., 2016), *Aureolaria*, *Buchnera*, and *Striga* (Frailey et al., 2018), as well as for numerous holoparasites, including *Aphyllon* (*Myzorrhiza*), *Epifagus*, *Boulardia*, *Cistanche*, *Conopholis*, *Orobanche*, *Phelipanche* and *Lathraea* (Wicke et al., 2013; Samigullin et al., 2016; Schneider et al., 2018). Analysis of these plastid genomes has allowed reconstruction of the history of gene loss and genome reconfiguration in this family, in the course of transition to a holoparasitic lifestyle, revealing a limited set of commonly retained genes (Wicke et al., 2013; Wicke et al., 2016).

**Figure 1:**
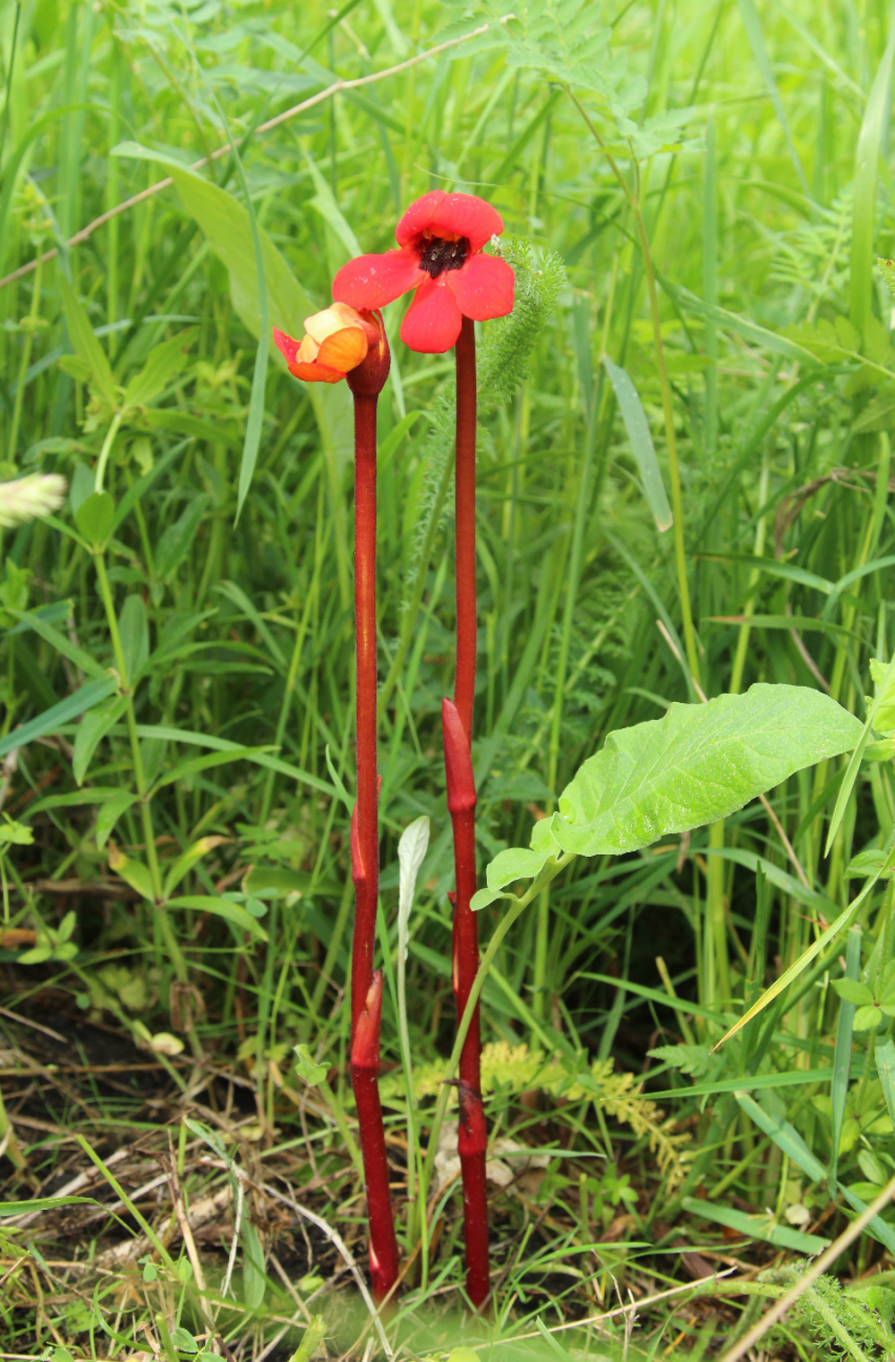
General view of *Diphelypaea coccinea* plants. Source credit: Vitaly V. Kadnikov.

The genus *Diphelypaea* (Nicolson, 1975), also known as *Phelypaea*, is phylogenetically close to *Orobanche* sect. *Orobanche* (Schneeweiss et al., 2004a) and comprises two species, *Diphelypaea coccinea* (M.Bieb.) Nicolson and *Diphelypaea tournefortii* (Desf.) Nicolson, which occur in the Caucasus, Crimea and Western Asia (Turkey and Iran). *D. coccinea* is an achlorophyllous obligately parasitic perennial herbaceous plant up to 30–50 cm in height. The stem is unbranched, red to reddish brown, ending in a single flower of a bright red color (Fig. 1). *D. coccinea* parasitizes the roots of plants of the genus *Psephellus*. In order to further explore plastome evolution in the course of transition to holoparasitism, and to clarify the phylogenetic position of *Diphelypaea*, we determined the complete sequence of the plastid genome of *D. coccinea* and compared it to previously sequenced plastomes of Orobanchaceae.

## Materials & Methods

### DNA extraction, genome sequencing and sequence assembly

The above ground portion of a single *D. coccinea* plant growing in a mountain region near the town of Kislovodsk, North Caucasus, Russia (43°50′59.8″N, 42°38′38.7″E) was used for the extraction of total genomic DNA, by a CTAB-NaCl method (Murray & Thompson, 1980). The extracted DNA was sheared using a g-TUBE device (Covaris Ltd., Brighton, UK) to obtain an average fragment size of 8 kb. The sheared DNA was then electrophoresed on a 0.5% agarose gel, and a portion of gel containing fragments of 6–9 kb was excised. The DNA was purified with a QIAquick Gel Extraction Kit (Qiagen, Hilden, Germany), and then used to prepare a long paired-end library according to the manufacturer’s instructions (Roche, Risch-Rotkreuz, Switzerland). The library was sequenced with a Roche GS FLX Genome Sequencer, using the Titanium XL+ protocol. About 329 Mb of sequence, with an average read length of 414 nts was generated. *De novo* assembly was performed with a Newbler Assembler v.2.9 (454 Life Sciences, Branford, CT, USA) with default settings, which yielded six plastid DNA contigs with an average coverage of 56-fold, ordered in a single scaffold. These contigs were identified based on sequence similarity to plastid genomes of Orobanchaceae and high coverage. The complete plastid genome sequence was obtained through generation of appropriate polymerase chain reaction (PCR) fragments spanning the junctions of the contigs and their sequencing by the Sanger method, using an ABI PRISM 3730 analyzer (Applied Biosystems, Waltham, MA, USA). The list of primers is available in Table S1. Reads spanning junctions between single copy regions and inverted repeats were used to infer contiguous sequences. To verify the correct assembly of the reconstructed plastid genome, raw reads were mapped against the reconstructed sequence with GS Reference Mapper (454 Life Sciences, Branford, CT, USA).

The raw reads were deposited in the Sequence Read Archive (SRA) under the accession number SRR9665263. The sequence of the plastid genome of *D. coccinea* was submitted to GenBank under accession number MK922354.

### Plastid genome annotation and analysis tools

Plastid genome annotation was performed using the Dual Organellar GenoMe Annotator (DOGMA; Wyman, Jansen & Boore, 2004), with further manual correction using similarity searches against previously annotated plastid genomes. The tRNAscan-SE server was also used to locate tRNA genes (Lowe & Chan, 2016). A circular map of the plastome was drawn using OrganellarGenomeDRAW software (Lohse et al., 2013).

Phylogenetic analysis was performed using concatenated nucleotide sequences of 17 conserved protein-coding genes (*matK*, *rpl14*, *rpl16*, *rpl2*, *rpl20*, *rpl33*, *rpl36*, *rps11*, *rps12*, *rps14*, *rps18*, *rps19*, *rps2*, *rps3*, *rps4*, *rps7*, *rps8*) from plastid genomes of 30 species of Orobanchaceae: *Aphyllon uniflorum* var. uniflorum (MH580290), *Aphyllon fasciculatum* (MH580292.1), *Aphyllon epigalium* subsp. epigalium (MH050785), *Aphyllon californicum* (syn. *Myzorrhiza californica*, NC_025651), *Boulardia latisquama* (HG514460), *Castilleja paramensis* (KT959111), *Cistanche deserticola* (KC128846), *Cistanche phelypaea* (HG515538), *Conopholis america* (HG514459), *Epifagus virginiana* (M81884), *Lathraea squamaria* (KM652488), *Lindenbergia philippensis* (HG530133), *Orobanche rapum-genistae* (KT387725), *Orobanche californica* (HG515539.2), *Orobanche cernua* var. cumana (KT387722), *Orobanche crenata* (HG515537), *Orobanche pancicii* (KT387724), *Orobanche austrohispanica* (KT387721), *Orobanche densiflora* (KT387723), *Orobanche gracilis* (HG803179), *Pedicularis ishidoyana* (KU170194), *Pedicularis hallaisanensis* (NC_037433), *Phelipanche purpurea* (HG515536), *Phelipanche ramosa* (HG803180), *Rehmannia piasezkii* (KX636160), *Rehmannia elata* (KX636161), *Rehmannia glutinosa* (KX636157), *Rehmannia solanifolia* (KX636159), *Schwalbea americana* (HG738866), and *Triaenophora shennongjiaensis* (MH071405). The plastid genome of *Nicotiana tabacum* (Z00044) was used as an outgroup. Nucleotide sequences were extracted from GenBank, concatenated for each of the genomes and aligned using MAFFT v.7.055b (Katoh & Standley, 2013), with default parameters. Poorly aligned regions were excluded using trimAl v.1.2rev59 software with the -gappyout option (Capella-Gutiérrez, Silla-Martínez & Gabaldón, 2009). A maximum likelihood phylogenetic tree was constructed using PhyML v.3.3 (Guindon et al., 2010). The Hasegawa-Kishino-Yano nucleotide substitution model with the gamma model of rate heterogeneity (HKY+Γ) was selected using jModeltest v. 2.1.10 (Posada, 2008).

### Verification of the presence of two copies of *clpP*)

To verify the presence of two copies of the *clpP* gene, we designed primer pairs flanking each copy (Table S1). Appropriate PCR fragments obtained using DNA samples extracted from two individual *D. coccinea* plants were analyzed by agarose gel electrophoresis (Fig. S1) and sequenced by the Sanger method.

## Results and Discussion

### Plastid genome structure and gene content

**Figure 2:**
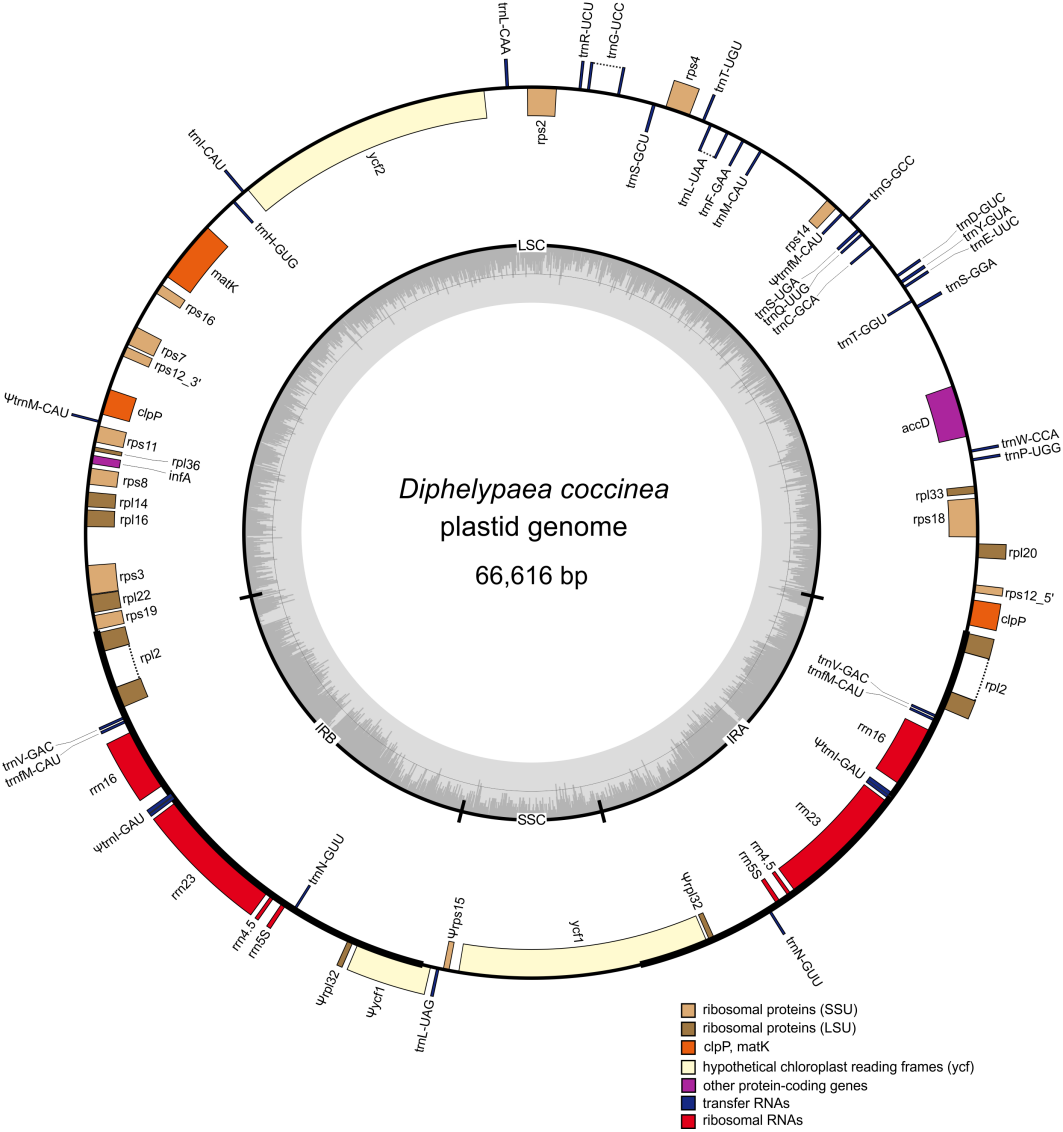
Gene map of the *D. coccinea* plastid genome. Genes shown inside the outer circle are transcribed clockwise, and those outside the circle are transcribed counter clockwise. Differential functional gene groups are color-coded. Pseudogenes are marked by *ψ*. GC content variation is shown in the middle circle.

The plastome of *D. coccinea* was assembled into a circular sequence of 66,616 bp from approximately 1.3 million paired-end reads (∼6 kb fragments). It has a typical quadripartite structure with a 37,964 bp large single copy (LSC) region, a 5,220 bp small single copy (SSC) region and a pair of inverted repeats (IRs), each of 11,716 bp (Fig. 2).

The *D. coccinea* plastome was predicted to contain 54 presumably intact unique genes (Table 1), which was fewer than its fully autotrophic relative *Lindenbergia philippensis* (113 genes), but comparable to that of holoparasitic Orobanchaceae (42–74; Wicke et al., 2013). Consistent with the inability to photosynthesize and the holoparasitic lifestyle of *D. coccinea*, its plastome lacks all genes coding for the NAD(P)H dehydrogenase complex and photosynthesis-related proteins. In particular, the *D. coccinea* plastome lacks the ATP synthase genes that are retained intact in most parasitic Orobanchaceae (Wicke et al., 2013). The genes for plastid-encoded RNA polymerase are also missing.

**Table 1:** Summary of genes identified in the *D. coccinea* plastome.

Function	Genes^a^
Ribosomal proteins (large subunit)	***rpl2***^*i*^*, rpl14,****rpl16****,****rpl20****, rpl22,****rpl33****,****rpl36***
Ribosomal proteins (small subunit)	***rps2****, rps3,****rps4****,****rps7****,****rps8****,****rps11****,****rps12***^*i*^*,****rps14****, rps16****, rps18****, rps19*
Other protein-coding genes	*infA,****matK****,****clpP****(2 genes), accD,****ycf1****,****ycf2***
rRNAs	***rrn16, rrn23***^b^, ***rrn4.5, rrn5***
tRNAs	*trnC-GCA,****trnD-GUC****,****trnE-UUC****, trnF-GAA, trnG-GCC, trnG-UCC*^*i*^*,****trnH-GUG****,****trnI-CAU****,****trnL-CAA****, trnL-UAA*^*i*^*,****trnL-UAG****,****trnM-CAU****,****trnfM-CAU****,****trnN-GUU****,****trnP-UGG****,****trnQ-UUG****, trnR-UCU,****trnS-GCU****, trnS-GGA, trnS-UGA, trnT-GGU, trnT-UGU, trnV-GAC,****trnW-CCA****,****trnY-GUA***
Pseudogenes	*ψ*rpl32, *ψ*rps15, *ψ*trnI-GAU ^i^, *ψ*trnfM-CAU, *ψ*trnM-CAU

**Notes.**

a Genes duplicated in inverted repeats were counted once, ^*i*^ denotes intron-containing genes, including trans-spliced *rps12.* Genes present in all Orobanchaceae species analysed in (Wicke et al., 2016) are shown in bold.

b *rrn23* gene contains 259-bp intervening sequence.

Most of the retained genes are involved in protein synthesis: four rRNA genes, 25 tRNA genes, seven genes coding for the small subunit ribosomal proteins and 11 for the large subunit ribosomal proteins. Most ribosomal protein genes usually found in plastid genomes of photosynthetic angiosperms were also in the plastome of *D. coccinea* (Table 1). Exceptions are *rps15* and *rpl32*, which are retained as truncated pseudogenes, and *rpl23,* which could not be identified. The loss of these genes has also been reported in other holoparasitic Orobanchaceae (Wicke et al., 2013). While the plastid genome of *D. coccinea* contains genes for 25 tRNA species (Table 1), it lacks the tRNA genes *trnA-UGC*, *trnI-GAU*, *trnK-UUU*, *trnR-ACG* and *trnV-UAC*, which are usually present in the plastomes of photosynthetic flowering plants. The loss of essential tRNA genes has been observed in parasitic plant plastomes showing an advanced stage of degradation (DePamphilis & Palmer, 1990; Delannoy et al., 2011; Wicke et al., 2013; Ravin et al., 2016). Analysis of the plastomes of holoparasitic Orobanchaceae revealed that up to 13 of 30 conserved tRNA genes, including those mentioned above, could be lost or pseudogenized (Wicke et al., 2013).

Besides genes involved in protein synthesis, the *D. coccinea* plastid genome contains genes *infA*, *matK*, *accD*, *clpP*, *ycf1* and *ycf2*, all of which were lost in some lineages of angiosperms, both parasitic and autotrophic. The most frequently lost gene is *infA*, encoding a translation initiation factor, the loss of which has been described in at least 24 separate lineages of angiosperms (Millen et al., 2001). The AccD protein, the beta subunit of acetyl-CoA carboxylase involved in fatty acid synthesis and leaf development (Kode et al., 2005), is essential for plastome maintenance (Krause, 2012). The gene for this protein is preserved even in the plastomes of most parasitic plants, although loss from the plastome and functional relocation to the nucleus occurs in some photosynthetic species (Rousseau-Gueutin et al., 2013). Among the Orobanchaceae, it has a 5′ truncation in the hemiparasite *Schwalbea americana* and holoparasitic species *Phelipanche purpurea* and *Phelipanche ramosa* (Wicke et al., 2013). Although *ycf1* and *ycf2* are considered to be essential for plastid maintenance (Drescher et al., 2000), multiple instances of loss of these genes, without transfer to the nuclear genome, have been reported in plant plastids (e.g., Wakasugi, Tsudzuki & M, 2001; Cai et al., 2008), but not in parasitic Orobanchaceae (Wicke et al., 2013). The functionality of the *ycf1* and *ycf2* genes in the *D. coccinea* plastome might be questioned, since their deduced protein products have long repeat-containing internal insertions contrary to typical Ycf1 and Ycf2 proteins, e.g., from *N. tabacum*. However, these insertions did not interrupt the open reading frames, suggesting that the proteins could retain functionality.

An interesting finding was the detection of two likely functional copies of the *clpP* gene in the *D. coccinea* plastome. *clpP* encodes a proteolytic subunit of Clp protease involved in protein metabolism within the plastid (Krause, 2012), and was proposed to be essential, being present even in highly reduced plastomes of parasitic plants (Delannoy et al., 2011; Ravin et al., 2016). Among the Orobanchaceae, a presumably functional *clpP* was found in all species (Wicke et al., 2013). In the *D. coccinea* plastome, two copies of *clpP* with 86% nucleotide sequence identity were found (Fig. 1). This duplication of the *clpP* gene was confirmed by PCR for two individual *D. coccinea* plants (Fig. S1). Notably, both genes lacked the introns usually present in *clpP* of photosynthetic angiosperms, but often absent in parasitic species. Their deduced protein products showed 75% amino acid sequence identity, but <35% identity with other plastidial ClpP proteins. It should be noted, that fast evolution of ClpP has been observed in several parasitic and photosynthetic lineages (Wicke et al., 2013; Sloan et al., 2014). Both gene copies contained intact reading frames; therefore, they probably remain functional.

Reduction of the size and gene content of the *D. coccinea* plastome is also reflected in the loss of introns in the remaining genes. Introns are only present in *rpl2*, *rps12* (*trans*-spliced), *trnG-UCC* and *trnL-UAA*, and appeared to be lost in *clpP*, *rpl16*, *rps16* and *rps12* (*cis*-spliced intron). The presence of *matK* correlates with the retention of a group IIA intron in the *rpl2* gene, which requires maturase activity for splicing (Zoschke et al., 2010).

As in most angiosperms, the *D. coccinea* plastome contained an *rrn* gene cluster within an IR region. Although the gene order is typical (*rrn16-rrn23-rrn4-rrn5*), two tRNA genes located in the *rrn16-rrn23* spacer were lost (*trnA-UGC*) or truncated as a pseudogene (*trnI-GAU*). Interestingly, the *rrn23* gene contains a 259 bp intervening sequence, absent from any other plastidial *rrn23* sequence available in the GenBank database. A BLASTn search against GenBank found no sequences with high similarity to this insert. The insert occurred in the side hairpin of the H38 helix region of domain II of the *rrn23* gene (Fig. S2). Introns in *rrn23* genes have been found in the plastomes of the charophytes *Chlorella* (Wakasugi et al., 1997) and *Chlamydomonas* (Turmel et al., 1993), and the hornwort *Anthoceros formosae* (Kugita et al., 2003); however, they have not been reported in other land plants. The site of insertion in the *D. coccinea rrn23* gene did not match the positions of intron insertions in the charophytes and *Anthoceros formosae*. It is possible that this intervening sequence is not an intron but an insertion that is still compatible with the final structure of the ribosome. RNA-seq analysis would help to clarify this issue.

### Structural rearrangements and duplications in the *D. coccinea* plastome

Plastid genomes of most angiosperms are highly conserved, not only in terms of overall quadripartite structure and gene content, but also in the gene order. Although deviation from a conserved gene order due to plastid genome rearrangements occurred in some photosynthetic lineages, e.g., cereals, geranium and clover (Chumley et al., 2006; Cai et al., 2008), numerous translocations, duplications, inversions and deletions are most frequently observed in the plastomes of parasitic species (Wicke et al., 2013). Among Orobanchaceae, the plastome of autotrophic *Lindenbergia philippensis* is colinear with that of tobacco, and limited deviations are observed in the hemiparasitic species *Schwalbea americana* (Wicke et al., 2013). In addition, no major rearrangements are observed in the plastome of *Lathraea squamaria*, belonging to the Rhinantheae clade of Orobanchaceae (Samigullin et al., 2016). However, gene deletions, duplications, inversions, shifts of IR boundaries and even complete loss of one IR are observed in the plastomes of holoparasitic species of the Orobancheae clade (Wicke et al., 2013; Schneider et al., 2018).

Comparison of the order of genes in the plastome of *D. coccinea* with the standard for angiosperms showed that the *D. coccinea* plastome, in addition to gene losses, experienced multiple rearrangements, including inversion, translocation and duplication of genes (Fig. 2). The gene order differs from that in other species of Orobanchaceae reported by Wicke et al. (2016). However, the *D. coccinea* plastome retains the highly conserved S10 operon (*rpl2*, *rps19*, *rpl22*, *rps3*, *rpl16*, *rpl14*, *rps8*, *infA*, *rpl36*, *rps11*) and the *rrn* gene cluster. These operons are conserved in all plastomes of Orobanchaceae, but appear to be deconstructed in some of the most highly reduced genomes of parasitic plants (Bellot & Renner, 2015; Ravin et al., 2016).

**Figure 3:**
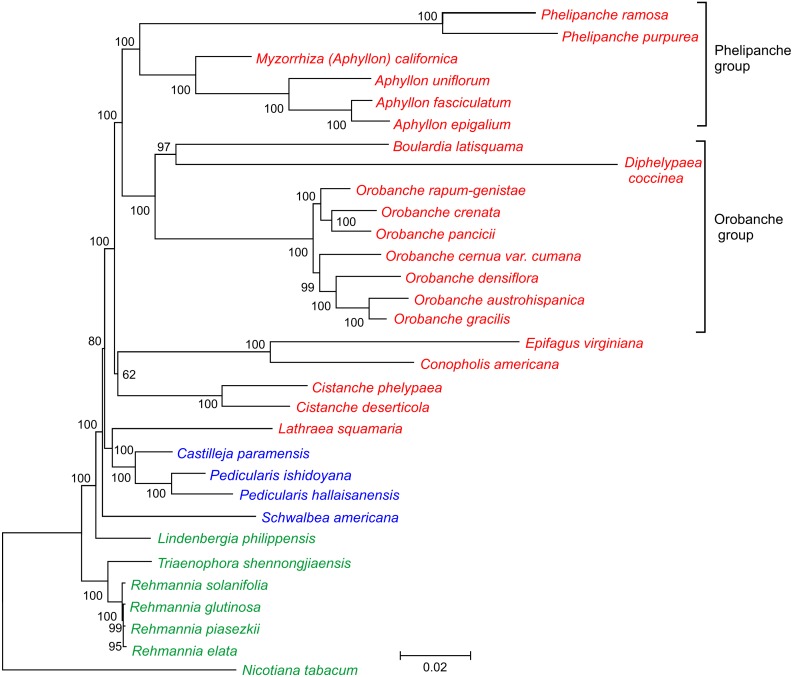
Phylogenetic tree of 30 taxa of *Orobanchaceae*. The tree was inferred by the maximum likelihood approach. Bootstrap support values are provided at the nodes. The scale bar corresponds to 0.02 substitutions per site. Autotrophic taxa are shown in green, hemiparasites in blue and holoparasites in red.

### Phylogenetic position of *D. coccinea*

Relatively little is known about the phylogenetic position of the genus *Diphelypaea*, and only two ∼600 bp-long nuclear internal transcribed spacer (ITS) sequences of *D. coccinea* and *D. tournefortii* are available in GenBank (accessed on May 3, 2019). Phylogenetic analysis of the nuclear ITS region revealed that the genus *Orobanche*, as defined by *Beck-Mannagetta (1930)*, divided into two genus-level groups, the *Orobanche* group (*Orobanche* sect. *Orobanche* and *Diphelypaea*) and the *Phelipanche* group (Schneeweiss et al., 2004a). This split is also supported by karyological features, since the chromosome base number in the *Orobanche*/*Diphelypaea* group is *x* = 19, while in the *Phelipanche* group it is *x* = 12 (Schneeweiss et al., 2004b). In the ITS phylogeny, two *Diphelypaea* species formed a basal lineage in the *Orobanche* group. However, these molecular phylogenetic implications were based on a limited sequence dataset and need to be clarified.

In this current study, we took advantage of availability of the complete plastid genome sequence of *D. coccinea* to define the phylogenetic position of this genus in the Orobanchaceae. Analysis of the concatenated nucleotide sequences of 17 conserved genes from 30 species of Orobanchaceae yielded a fully resolved phylogenetic tree (Fig. 3). *D. coccinea* appeared to be included in a cluster also comprising *Boulardia latisquama* and species of *Orobanche* sect. *Orobanche*. By contrast, the *Phelipanche* group, comprising *Phelipanche* and *Aphyllon*, formed a distinct lineage.

## Conclusions

Being only 66,616 bp in size and containing 54 presumably intact unique genes, the plastome of *D. coccinea* is one of the most highly reduced among the parasitic Orobanchaceae. Plastome rearrangements, gene duplications and the loss of introns are associated with gene loss and genome reduction. More pronounced gene loss has only been reported in the plastomes of *Conopholis americana* (45,673 bp, 42 genes), *Epifagus virginiana* (70,028 bp, 42 genes) and *Boulardia latisquama* (80,361 bp, 49 genes). In particular, the *D. coccinea* plastome lacks all genes of the photosynthetic apparatus, including ATP synthase genes that are retained intact in most Orobanchaceae. However, all 16 protein-coding genes, 14 tRNA genes and 4 rRNA genes commonly present in the plastid genomes of all hemi- and holoparasitic Orobanchaceae species (Table 1) are present in *D. coccinea*, suggesting that further gene loss is unlikely in this lineage. Phylogenetic analysis confirmed that *D. coccinea* belongs to the *Orobanche* group of the family Orobanchaceae.

##  Supplemental Information

10.7717/peerj.7830/supp-1 Figure S1 Confirmation of the duplication of the *clpP* gene via PCR Agarose gel electrophoresis of PCR fragments obtained with primer pairs DCLP1F/DCLP1R (lanes 1 and 3) and DCLP2F/DCLP2R (lanes 2 and 4). DNA samples isolated form two individual plants (plant 1–lanes 2 and 3, plant 2 –lanes 4 and 5) were used as templates for amplification. Lane 1,—molecular weight marker (sizes are shown in kb). Expected lengths of PCR fragments: *clpP* copy 1 (primers DCLP1F and DCLP1R) –772 bp; *clpP* copy 2 (primers DCLP2F and DCLP2R) –949 bp

10.7717/peerj.7830/supp-2 Figure S2 Predicted secondary structure of the 23S rRNA and intervening sequence Sequence and structure of the intervening sequence in *D. coccinea* 23S rRNA is shown in the left part. The secondary structure was predicted by the RNAFold Web Server ( http://rna.tbi.univie.ac.at//cgi-bin/RNAWebSuite/RNAfold.cgi) using minimum free energy fold algorithm. The right part shows the nucleotide sequence and secondary structure of the domain II of the chloroplast 23S rRNA from *Spinacia oleracea* (modified from Bieri et al., EMBO J. 2017; 36(4): 475–486). The 23S rRNA is 95% identical in sequence between the *D. coccinea* and the spinach suggesting similar secondary structures. In the *D. coccinea* 23S rRNA the intervening sequence is located at a position corresponding to nucleotide 951 in the spinach 23S rRNA sequence.

10.7717/peerj.7830/supp-3 Table S1 Primers used for plastome assembly and confirmation of the duplication of the *clpP* gene PCR amplifications were performed on Mastercycler personal PCR machine (Eppendorf) using 1.25 units GoTaq^®^ DNA Polymerase (Promega) per 50 µl amplification reaction in the Green GoTaq^®^ Reaction Buffer supplemented with 0.2mM each dNTP and 1.0 µM each primer. Cycling conditions were as follows: 96 ° C for 5 minutes, followed by 40 cycles of 96 ° C for 40″, 50 ° C for 60″and 72 ° C for 60″, and a final 5 minute elongation step at 72 ° C.

## Additional Information and Declarations

### Data Availability

The following information was supplied regarding data availability: The raw reads are available in the Sequence Read Archive (SRA): SRR9665263 and the sequence of the plastid genome of *D. coccinea* is available at GenBank: MK922354.
