# Supplementary figures and images for: Extensive plastome reduction and loss of photosynthesis genes in Diphelypaea coccinea, a holoparasitic plant of the family Orobanchaceae
Source: PeerJ. 2019 Oct 2;7:e7830. doi: 10.7717/peerj.7830 (PMC6778433; doi:10.7717/peerj.7830)

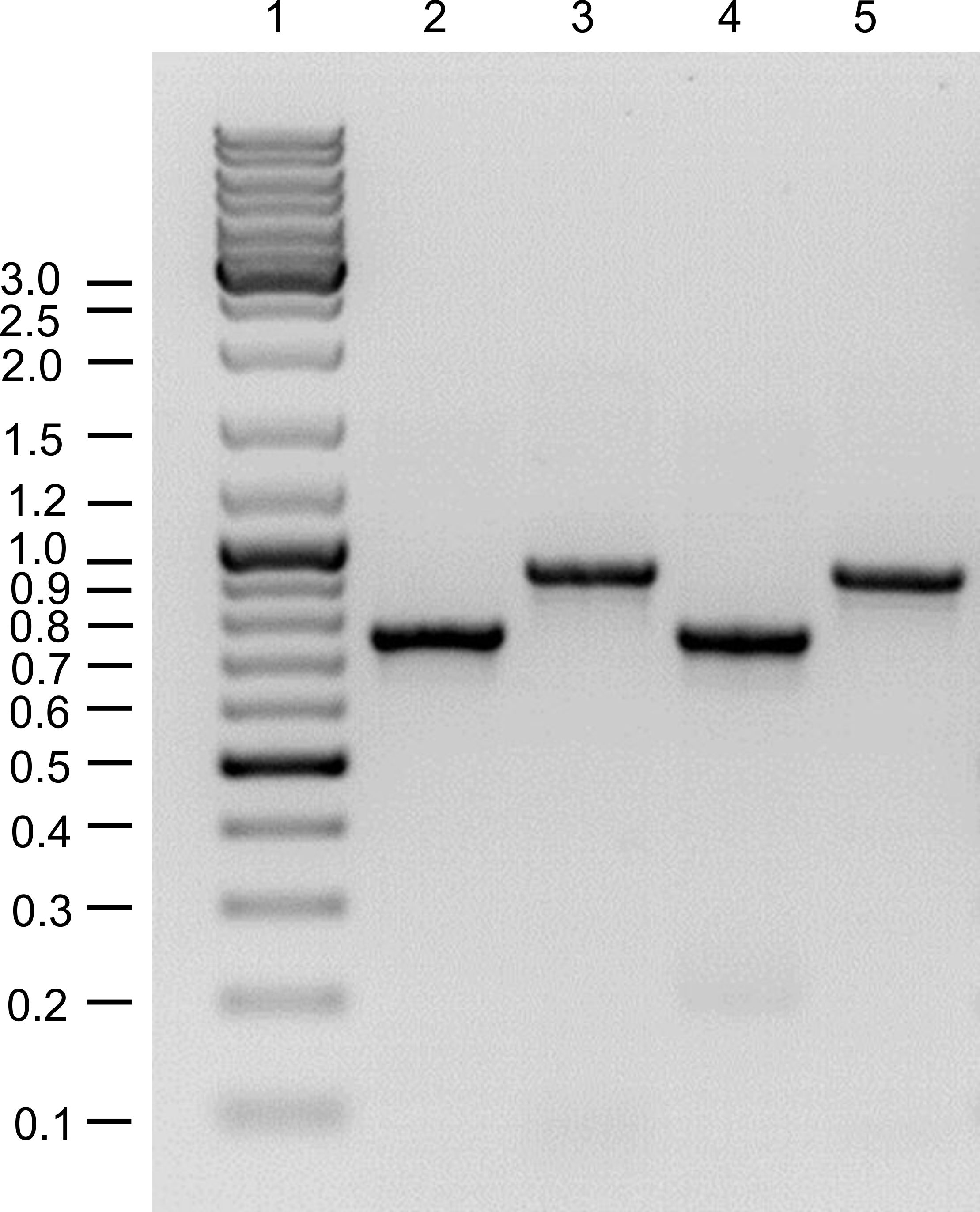

Supplement: Figure S1 — Agarose gel electrophoresis of PCR fragments obtained with primer pairs DCLP1F/DCLP1R (lanes 1 and 3) and DCLP2F/DCLP2R (lanes 2 and 4). DNA samples isolated form two individual plants (plant 1–lanes 2 and 3, plant 2 –lanes 4 and 5) were used as templates for amplification. Lane 1,—molecular weight marker (sizes are shown in kb). Expected lengths of PCR fragments: clpP copy 1 (primers DCLP1F and DCLP1R) –772 bp; clpP copy 2 (primers DCLP2F and DCLP2R) –949 bp [file peerj-07-7830-s001.jpg]

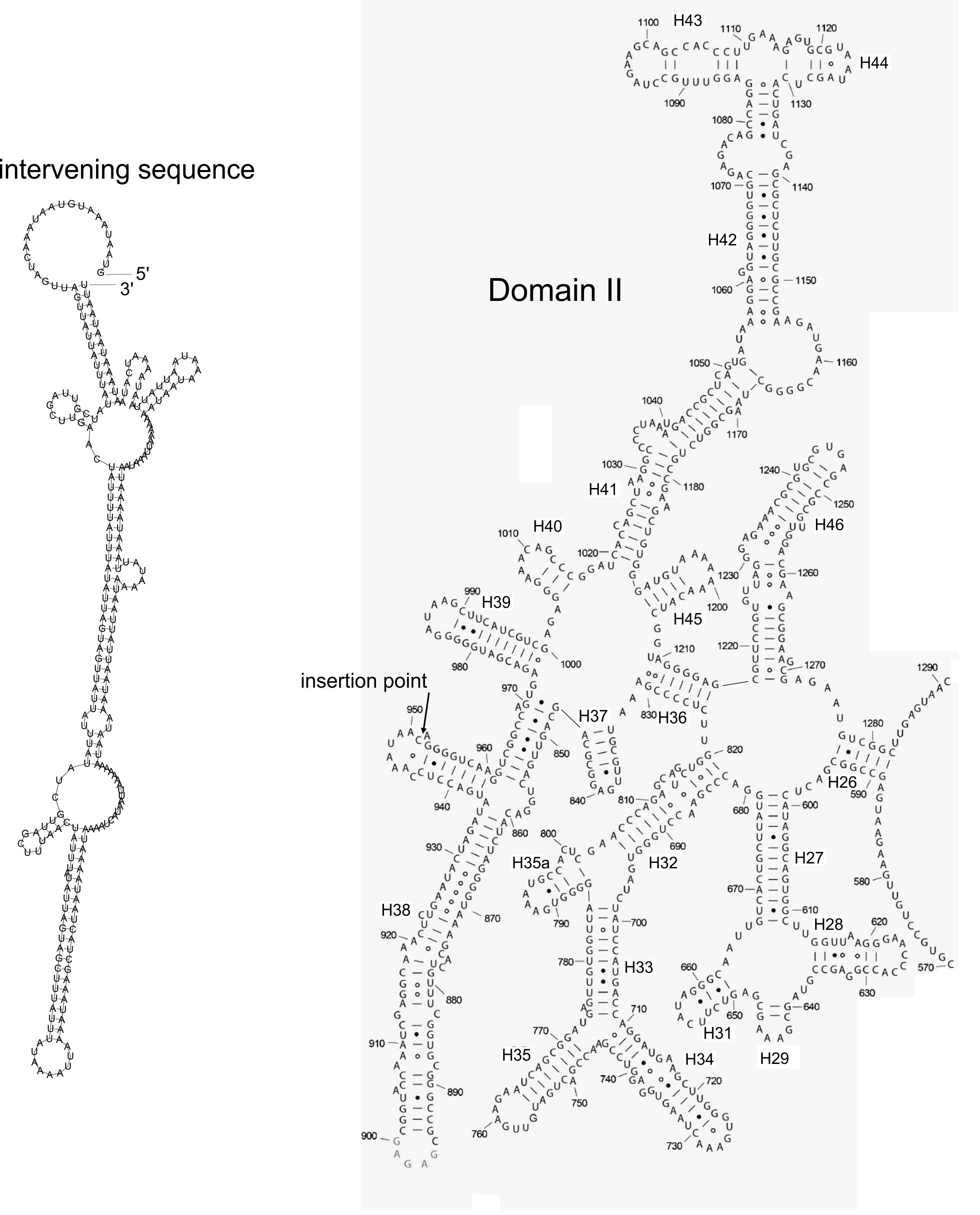

Supplement: Figure S2 — Sequence and structure of the intervening sequence in D. coccinea 23S rRNA is shown in the left part. The secondary structure was predicted by the RNAFold Web Server ( http://rna.tbi.univie.ac.at//cgi-bin/RNAWebSuite/RNAfold.cgi) using minimum free energy fold algorithm. The right part shows the nucleotide sequence and secondary structure of the domain II of the chloroplast 23S rRNA from Spinacia oleracea (modified from Bieri et al., EMBO J. 2017; 36(4): 475–486). The 23S rRNA is 95% identical in sequence between the D. coccinea and the spinach suggesting similar secondary structures. In the D. coccinea 23S rRNA the intervening sequence is located at a position corresponding to nucleotide 951 in the spinach 23S rRNA sequence. [file peerj-07-7830-s002.jpg]
