# Supplementary material for: Extensive plastome reduction and loss of photosynthesis genes in Diphelypaea coccinea, a holoparasitic plant of the family Orobanchaceae
Source: PeerJ. 2019 Oct 2;7:e7830. doi: 10.7717/peerj.7830 (PMC6778433; doi:10.7717/peerj.7830)
Supplement: Table S1 — PCR amplifications were performed on Mastercycler personal PCR machine (Eppendorf) using 1.25 units GoTaq® DNA Polymerase (Promega) per 50 µl amplification reaction in the Green GoTaq® Reaction Buffer supplemented with 0.2mM each dNTP and 1.0 µM each primer. Cycling conditions were as follows: 96 ° C for 5 minutes, followed by 40 cycles of 96 ° C for 40″, 50 ° C for 60″and 72 ° C for 60″, and a final 5 minute elongation step at 72 ° C. [file peerj-07-7830-s003.doc]

| Name | Sequence | Localization | Purpose |
| --- | --- | --- | --- |
| CDC_399R_1 | AAGTGATTGAGTTCAGTAGTTACTC | 40573-40549 | plastome assembly |
| CDC_1328R | TTCCAGGTATGATTTCTATGTTAT | 39596-39619 | plastome assembly |
|  |  |  |  |
| CDC_263R | GATAAAAGGTCCTGGTATAGGA | 34379-34358 | plastome assembly |
| CDC_262F | AAGTAGAGCTGCTGTTCCTATAA | 33663-33685 | plastome assembly |
|  |  |  |  |
| CDC_472F | TCTCATTGTAAAAGATCTGTTACTAAATA | 2567-2596 | plastome assembly |
| CDC_995R | AAGACAGTTCCTATTTGATATAGATA | 3243-3219 | plastome assembly |
|  |  |  |  |
| DCLP1F | TACCAATGATTTTATACCAGTGATTCT | 33287-33313 | *clpP* amplification |
| DCLP1R | ATCCCTACCCATAAGAAAAACTCTAT | 34058-34033 | *clpP* amplification |
|  |  |  |  |
| DCLP2F | TAAGCTATGATACCTTTACTAAATTACT | 1183-1210 | *clpP* amplification |
| DCLP2R | GCTGTCTTGTATTTCTAATAAGTTGAT | 2131-2105 | *clpP* amplification |
